# Prospective cohort study of voice outcomes following secondary tracheoesophageal puncture in gastric pull-up reconstruction after total laryngopharyngoesophagectomy

**DOI:** 10.1186/s40463-021-00492-3

**Published:** 2021-03-17

**Authors:** Emily C. Deane, Harman Parhar, Linda Rammage, Amanda Hu, Donald W. Anderson

**Affiliations:** 1 Division of Otolaryngology Head & Neck Surgery, Department of Surgery, University of British Columbia, 4th Floor, 2775 Laurel Street, Vancouver, BC V5Z1M9 Canada; 2 School of Audiology & Speech Sciences, University of British Columbia, Vancouver, Canada

**Keywords:** Gastric pull-up, Laryngopharyngoesophagectomy, Voice, Quality of life

## Abstract

**Background:**

Gastric pull-up is a reconstructive option for circumferential defects after resection of advanced laryngopharyngeal malignancy. Voice loss is expected and vocal rehabilitation remains a challenge. Our study objectives were to investigate the feasibility of secondary tracheoesophageal puncture following gastric pull-up and to analyze voice outcomes.

**Methods:**

This was a prospective cohort study of patients with advanced laryngopharyngeal malignancies who underwent gastric pull-up and secondary tracheoesophageal puncture between 1988 and 2017 at a tertiary-care academic institution. Objective acoustic measures included fundamental frequency and vocal intensity. Perceptual analysis was performed using voice recordings (“Rainbow Passage”) randomly presented in a blinded fashion to four clinicians using the validated GRBAS scale. Speech intelligibility was assessed in a blinded fashion using a validated 7-point scale. Additionally, the Voice Handicap Index-10 was administered as a validated patient self-reporting tool.

**Results:**

Ten patients (7 male, 3 female) were included, all of whom preferentially used tracheoesophageal puncture for communication. These patients had abnormal median fundamental frequency of 250 (interquartile range (IQR) 214–265) Hz and a limited median vocal intensity of 65.8 (IQR 64.1–68.3) dB. Perceptual analysis (GRBAS) revealed a median ‘moderate’ degree of impairment [grade 2 (IQR 2–3), roughness 2 (IQR 2–3), breathiness 3 (IQR 2–3), asthenia 2 (IQR 1–2), strain 2 (IQR 1–2)] as did median intelligibility scores [median 5 (IQR 4–7)]. Most patients self-reported an abnormal voice handicap-10 [median 26.5 (IQR 22.8–35.0)].

**Conclusion:**

Secondary tracheoesophageal puncture is a safe and feasible option for voice rehabilitation after gastric pull-up. Although analyses demonstrated moderate subjective and objective impairment, tracheoesophageal puncture provided patients with a self-reported means of functional verbal communication and was their preferred method of communication.

**Graphical abstract:**

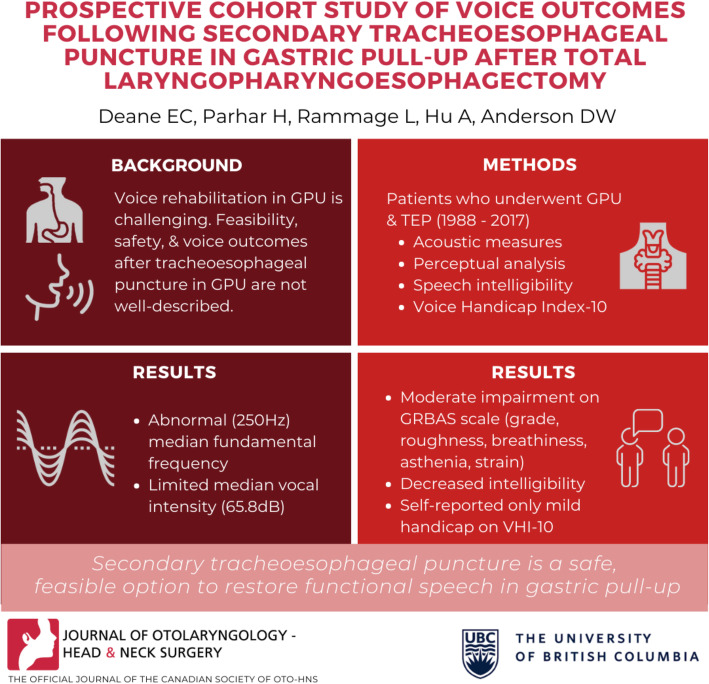

## Background

Gastric pull-up (GPU) is a reconstructive technique for circumferential defects following resection of advanced laryngopharyngeal malignancies [1]. With the popularization of free tissue transfers in the 1980s, however, many surgeons opted to use free tissue cutaneous flaps to reconstruct these defects in lieu of GPU [2, 3].

Tracheoesophageal puncture (TEP), first described in 1983 by Singer et al., has become widely adopted for voice rehabilitation following total laryngectomy (TL) and has demonstrated satisfactory voice outcomes [4–6]. In addition, TEP has also been demonstrated to be feasible following total laryngopharyngectomy where free flap reconstruction was performed [5]. The TEP procedure involves creating a fistula between the trachea and esophagus to establish a communication between the airway and the reconstructed pharyngoesophageal segment. A one-way valve prosthesis is placed in the puncture; occlusion of the tracheostoma during exhalation induces air shunting into the esophageal reservoir causing the tissue to vibrate, thereby creating the substitute voice source [7]. The current literature is limited to only a few case reports or small series describing the feasibility and safety of a TEP and prosthesis placement for the purposes of voice rehabilitation in GPU [8–12]. Noel et al. performed the most recent and largest *(n = 4)* study but their analyses included only subjective patient reported outcomes of voice quality [12].

Voice rehabilitation following GPU remains challenging for a variety of reasons, primarily because very few centers have a large volume of experience and because the acoustic properties of gastric mucosa are different than tissues for which TEP is generally performed following TL. At our institution, the primary investigator has performed TEP in GPU patients for the past approximately 20 years. The objectives of this study were to assess the feasibility and safety of TEP following GPU, to determine whether patients who underwent GPU with TEP ultimately used their prosthesis for ongoing voice generation, and to subjectively and objectively analyze the quality of the voice generated.

## Methods

### Cohort selection

Records for consecutive patients with advanced laryngopharyngeal malignancies requiring circumferential reconstruction who underwent GPU and TEP between 1988 and 2017 at the University of British Columbia performed by a single head and neck surgeon (DWA) were retrospectively reviewed. Secondary tracheoesophageal puncture was offered to interested patients at approximately 4 months after the index surgery. By this point, patients had time to recovery from surgery, complete any adjuvant therapy if required, and in most cases became accustomed to full oral intake without need for jejunostomy. Patients’ demographic data, medical history, and time from original surgery to voice prosthesis insertion were extracted. Clinical covariates of interest were chosen, a priori, and included: age, sex, site of primary malignancy, time to TEP placement, and history of head and neck irradiation. Voice analyses and collection of patient-reported vocal quality of life were collected prospectively in surviving patients starting in 2019.

### Procedure details

Procedural details for the ablation and reconstruction have been previously described by Butskiy et al. 2020 [13]. In brief, all included patients underwent an en bloc laryngopharyngectomy performed by the otolaryngology team. The thoracic surgery team specific to each hospital was responsible for thoracic esophagectomy (when required) and gastric mobilization, performed through an exclusively transhiatal approach without thorascopic guidance and without need for thoracotomy. A well-vascularized and tensionless gastropharyngeal anastomosis was subsequently performed using interrupted Connell sutures [14]. All patients underwent concurrent jejunostomy tube placement and received an electrolarynx for short-term voice rehabilitation prior to discharge from hospital. Secondary tracheoesophageal puncture was offered to interested patients at approximately 4-months after the index surgery. The only absolute contraindication to TEP was patient fitness. Their perceived teachability and ability to present themselves for follow-up was a relative contraindication. The decision to proceed with TEP placement was largely informed by the patient’s own preferences.

All secondary TEP procedures were performed under general anesthesia with intermittent ventilation provided with a cuffed endotracheal tube inserted into the existing laryngectomy stoma. Cervical rigid esophagoscopy was performed using a short scope to visualize the neopharynx. The scope was oriented with the bevel facing anteriorly to allow for transillumination through the posterior tracheal wall. The endotracheal tube was temporarily removed after preoxygenation. A puncture site 8 mm below the mucocutaneous junction was generally selected. Early on in the study*,* a red rubber catheter was placed through the puncture site and the patients were then fitted with a prosthesis by our speech language pathologist. After 2018, the commercially available Provox kit (Atos Medical, Horby, Sweden) was utilized using a modified Seldinger technique designed for traditional laryngectomy patients [15]. In brief, a sharp trocar with cannula was used to create a puncture towards the lumen of the scope at the desired site under direct visualization. The cannula was oriented superiorly to facilitate the passing of the provided flexible catheter superiorly exiting the oral cavity. The cannula and esophagoscope were then removed. A voice prosthesis was attached to the oral tip of the catheter which was then pulled through the stoma in a retrograde fashion allowing its placement in the fistula between the trachea and neoesophagus. A hemostat was often required to pull the flange through the fistula and allow for final positioning. The introduction attachment connected the prosthesis to the catheter was then cut and the ventilation was resumed. Once awake, the prostheses were tested to ensure impermeability of liquids into trachea.

### Voice analyses

Voice recordings were made of patients reciting a standardized passage*.* The “Rainbow Passage” is phonetically balanced for the English language and used frequently in voice outcomes research [16]. Voice samples were recorded in a quiet room with a professional portable recorder (H4N Pro, Zoom, Hauppauge, NY, US) held 10 cm from the patient’s stoma. Voice samples were stored on removable hard drives for blinded review. Dedicated software [Visi-Pitch IV, model 3950 B (Pentax Medical, Montvale, NJ US)] was utilized for objective voice analysis. Specifically, fundamental frequency (f_0_, lowest frequency, first harmonic of the sound wave resulting from vibration of the gastric tissue) and vocal intensity (loudness, dB [17]) were obtained from sustained phonation of /i/ and pitch glide of at least two attempts. It should be noted that f_0_ from male and female subjects were pooled for analyses as previously done in related literature given that the sound source (neopharyngeal gastric mucosa) is similar in both groups [18].

Subsequently, blinded perceptual analysis of voice quality was performed using the validated GRBAS tool, introduced by the Committee for Phonatory Function Tests of the Japan Society of Logopedics and Phoniatrics [19]. Voice samples were randomly presented to four trained clinicians: a fellowship trained laryngologist, a speech language pathologist with over 40 years of experience in voice, and two otolaryngology residents. Grade, breathiness, roughness, asthenia and strain were scored on a scale from 0 to 3. Recordings were played twice before raters were asked to assign a score. Each clinician duplicated voice sample ratings in a random blinded manner to calculate intra-rater reliability. Intelligibility assessment was performed by a group of 10 non-otolaryngologic health care professionals in a blinded fashion using a validated 7-point scale [20], where 1 denotes no noticeable differences from normal, and 7 denotes unintelligible speech. Raters were each played a recording from a single patient in a quiet room and were then asked to assign a score.

### Patient-reported outcomes

Patient-reported outcomes were assessed using the *Voice Handicap Index-10* [21], a validated, self-reported questionnaire that quantifies the degree of handicap a patient experiences from his/her/their voice using a 10-item survey. The total score has a range of 0 to 40, where a score of 11 or greater is considered abnormal. In all included cases, the VHI-10 was administered prospectively to patients after a minimum of 1 year of TEP prosthesis use to avoid bias related to the learning curve.

### Statistical analyses

All clinical data were de-identified, tabulated and compiled for statistical review, which was performed in SPSS Version 25 (IBM, Armonk, NY). Descriptive statistics were performed using non-parametric techniques to account for the non-normally distributed voice outcomes. Fleiss’ Kappa was used to calculate the inter-rater agreement between 4 raters for each one of the 5 measures on the GRBAS scale. Unweighted Cohen’s Kappa was used to determine the index of intra-rater agreement within raters in an unweighted manner. The datasets used and analysed are available from the corresponding author on reasonable request.

## Results

There have been a total of 49 patients who underwent GPU at our institution between 1988 and 2019 by the principal investigator. After 2005, all patients who underwent GPU were provided with an electrolarynx after surgery and then were offered the opportunity to undergo TEP insertion at approximately 4 months post-operatively. Twenty-four patients underwent GPU between 2005 and 2019 with DWA and 12 had secondary TEP placement. Two of these patients died between the time of TEP placement and the period of voice assessment for the purposes of this study, leaving 10 included patients (Table 1). The cause of death for the 2 patients not included was disease recurrence; they had no known complications of their TEPs.

**Table 1:** Patient demographics of the study population (*n* = 10)

ID	Age	Sex	Site	Months to TEP	Prior XRT
1	47	M	Lx	10.5	yes
2	54	M	HPx, Lx, Tr, Th	12.1	no
3	81	M	Th	8.4	yes
4	64	M	HPx	--	yes
5	40	F	HPx	4.4	yes
6	63	M	HPx	--	no
7	69	M	HPx	12.7	yes
8	67	F	HPx, E	9.0	yes
9	60	M	HPx, E	--	yes
10	62	F	Opx, Lx	3.8	yes

‘- -' = data missing. Sites: *HPx* hypopharynx, *Lx* larynx, *Opx* oropharynx, *Tr* tracheal, *E* esophageal, *Th* thyroid

The majority of the included patients were male (seven, 70.0%) and had primary tumors that involved the hypopharynx (seven, 70.0%). The median age was 60.7 (IQR 56.0–66.0) years at time of TEP placement and voice prostheses were placed after a median of 271.0 (IQR 192.0–338.0) days. There were no complications of TEP insertion. Eight (80.0%) patients had undergone radiation therapy prior to prosthesis placement, although only one had prior chemotherapy. At the time of post-operative assessment, all 10 (100.0%) patients preferentially used their prosthesis for communication.

The median fundamental frequency was 250.0 (IQR 214.0–265.0) Hz and vocal intensity 65.8 (IQR 64.1–68.3) dB – both of which would be considered abnormal using historical normative standards that were created using populations with intact laryngeal voice [22] (Table 2). Perceptually, patients’ voice quality was moderately to severely impaired in overall grade [2.0 (IQR 2.0–3.0)], roughness [2.0 (IQR 2.0–3.0)], breathiness [3.0 (IQR 2.0–3.0)], asthenia [2.0 (IQR 1.0–2.0)] and strain [2.0 (1.0–2.0)] (Table 2). Inter-rater agreement was substantial for grade (0.71, *p* < 0.01), fair for roughness (0.24, *p* < 0.01) and slight for breathiness (0.13, *p* = 0.15), asthenia (0.13, *p* = 0.15), and strain (0.01, *p* = 0.88) across the four expert raters. Intra-rater agreement by rater 1 was substantial (0.65, *p* < 0.01), 2 and 4 were moderate (0.46 and 0.45 respectively, *p* < 0.01) while rater 3 had slight intra-rater agreement (0.15, *p* = 0.10). Most patients reported an abnormal Voice Handicap Index-10 score (median 26.5 (IQR 22.8–32.0) (Table 2).

**Table 2:** Voice outcomes: Acoustic analysis, perceptual analysis, intelligibility scores, and patient-reported scores

ID	Acoustic Analysis				Perceptual Analysis					Intelligibility Score	Patient-reported Score
	f_o_ (Hz)	Vocal intensity (dB)	Min (dB)	Max (dB)	Grade Median (IQR)	Roughness Median (IQR)	Breathiness Median (IQR)	Asthenia Median (IQR)	Strain Median (IQR)		VHI–10 (0 – 40)
1	216	63.8	44.3	79.7	2 (0)	3 (1)	2 (1.3)	1 (0.3)	2 (0.3)	5	33
2	211	65.1	63.8	79.7	2 (0)	2 (0)	3 (1)	1 (0.5)	2 (0)	4	27
3	CNR	65.9	47.6	67.9	3 (0)	3 (0)	3 (0)	2 (0.3)	2.5 (1.3)	5	25
4	275	68.5	67.8	78.6	3 (0)	3 (1)	3 (0.3)	1 (0.5)	2 (2)	6	34
5	CNR	67.5	48.5	68.3	2 (0)	1 (1)	3 (0.3)	3 (1)	1 (0.5)	5	35
6	CNR	65.7	44.3	82.5	3 (0)	2 (1)	3 (0)	3 (1)	2 (2)	7	22
7	254	69.4	73.5	80.6	2 (0)	2 (0)	1 (0.5)	1 (0)	1.5 (1)	3	8
8	170	69.5	47.5	79.3	2 (0)	3 (0)	1 (0.5)	1 (0.3)	2 (0.3)	3	14
9	292	63.8	57.1	69.4	3 (0)	3 (0)	3 (1)	2 (1.3)	1.5 (1.3)	7	29
10	250	54	54	69.2	2 (0)	0 (1)	3 (0.3)	3 (1)	1.5 (1)	4	26
Overall [Median (IQR)]	250.0^a^ (214.0-265.0)	65.8 (64.1-68.3)	51.3 (47.5-62.1)	79.0 (69.3-79.7)	2 (2-3)	2 (2-3)	3 (2-3)	2 (1-2)	2 (1-2)	5 (4-7)	26.5 (22.8-32.0)

*f*_*o*_ Fundamental frequency, *CNR* Could not record, *VHI-10* Voice Handicap Index-10

^a^Based on *n* = 7 as it was not possible to record this in 3 patients

**Fig. 1:**
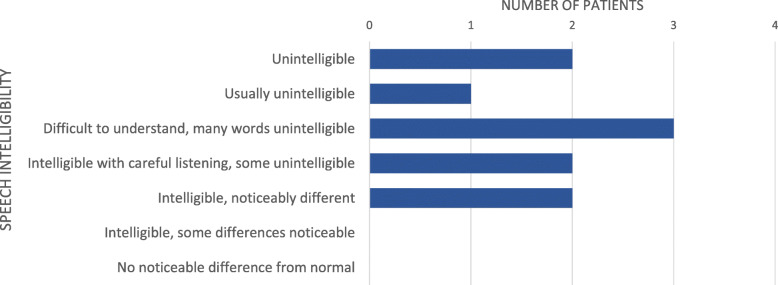
Speech Intelligibility Evaluation

A blinded panel of non-otolaryngology medical personnel found GPU patients’ speech difficult to understand with many words unintelligible based on a median score of 5.0 (IQR 3.0–7.0) on a 7-point scale (Table 2; Fig. 1).

## Discussion

In this paper, we describe a prospective cohort following GPU for circumferential defects of the laryngopharynx who underwent secondary TEP. All preferentially used TEP for communication. They had abnormal median fundamental frequency and a limited median vocal intensity. Perceptual analysis using the GRBAS tool revealed a median ‘moderate’ degree of impairment as did median intelligibility score. Most patients self-reported an abnormal Voice Handicap Index-10 score.

Gastric pull-up is among the most historically well-established techniques that are in use today for pharyngoesophageal junction reconstruction following oncologic resection [1]. Early enthusiasm for the use of GPU waned due to high mortality rates as well as with the development of alternative free tissue reconstructive procedures [2, 23, 24]. Recent systematic review data from Butskiy et al. demonstrates, however, that the mortality and morbidity of GPU among more contemporary cohorts is similar to alternative methods of pharyngoesophageal reconstruction, including microvascular-based techniques [25]. For many centers that have experience with GPU, this has led to a renewed interest in studying the functional outcomes that can be achieved with this operation including voice quality and communicative ability.

Esophageal speech, tracheoesophageal speech and electrolarynx speech are rehabilitation methods after total laryngectomy. Based on acoustic and perceptual outcomes, tracheoesophageal speech has been established as the favored speech form in TL [18], however, no preferred rehabilitation has been established for GPU. In our cohort, all patients used TEP as a primary means of communication and there were no complications after insertion, supporting the safety and feasibility of this technique in the GPU population. Secondary TEP in GPU has been referred to as “tracheogastric puncture”, “tracheogastric fistula” or “TGP” by some authors [9, 26], understandably because some patients had esophagectomies and the puncture was placed in gastric mucosa. The majority of authors in the literature [8, 10–12] though, still referred to this procedure as tracheoesophageal puncture (TEP). To be consistent with the literature, we used the term TEP.

The majority (80%) of patients in our study had a primary diagnosis of hypopharyngeal carcinoma and a majority (80%) occurred in the context of prior head and neck irradiation. Hypopharyngeal cancers are known to present at late stages due to a lack of early symptomatology and have a historically poor prognosis [27] as do cancers of the upper aerodigestive tract which recur in irradiated fields [28]. Studies looking at quality of life in patients treated for hypopharyngeal or cervical esophageal carcinomas are sparsely available, which may be due to its low incidence and/or its poor survival. However, findings from advanced aerodigestive tract cancers like laryngeal cancer may be generally applicable, as they face similar rehabilitative challenges. In a cohort of 48 patients, our center previously showed that GPU can facilitate rapid return to swallowing, with long-term survivors reporting relatively normal deglutition, as well as moderate levels of overall health and quality of life [13]. Despite this, rehabilitation of speech was found to be a significant challenge. In a subset of 10 patients who had secondary TEP after GPU, EORTC QLQ-H&N35 questionnaire results revealed a moderate degree of speech problems. Voice Handicap Index [29] administration resulted in a mean score of 62 (range 25–114) out of 120, which was considered moderate to severe. The paucity of other studies on the functional outcome of voice in this population inspired us to prospectively evaluate voice outcomes in GPU patients in a more rigorous manner.

Voice is a multidimensional construct which requires multiple modalities of assessment, namely subjective perceptual measures, objective acoustic analysis, and patient self-reported outcome measurements. A systematic review by Van Sluis and colleagues [18] saw that studies on speech outcomes after TL have been flawed in design and represent weak levels of evidence; they reiterated that is there is no standardized means of evaluating voice in substitute voice speakers [29]. In GPU, the transferred stomach is more patulous than the vibrating pharyngoesophageal segment in either esophageal or tracheoesophageal speech [9], and even those techniques are known to produce more noise components and less regularity than laryngeal voice [30]. Our acoustic analysis of GPU with secondary TEP demonstrated low vocal intensity which is in keeping with previous studies of alaryngeal speech [8, 30–34], and in particular, tracheoesophageal speech in other methods of circumferential reconstruction (e.g. tubed cutaneous free flap, jejunal grafts, etc) [5, 8, 35, 36]. Interestingly, our median pooled f_0_ was higher than esophageal, tracheoesophageal and even native laryngeal speakers subjects [8, 18, 34]. This confirmed to us that fundamental frequency may not be the most reliable measurement in this population, as has been previously reported in the literature [18]. Indeed, 30% of participants in our study were unable to produce a fundamental frequency result. Overall, the voice samples had high perturbation measures which also prevented the calculation of jitter and shimmer values for most patients.

Rigorous perceptual evaluation of voice is inherently challenging, further still with substitute voice users [37]. The GRBAS protocol has been established as a reliable tool [38] though is not widely used in alaryngeal patients. Certain authors have discouraged its use for substitute voicing as irregular voicing using alaryngeal techniques might consistently get scored as the highest grade of dysphonia on the GRBAS scale [39]. To avoid bias, recordings were de-identified, randomly presented, and played twice before our raters were asked to assign a score. Inter and intra-rater reliability were calculated for all GRBAS scores. GPU patients scored between grade ‘2’ and grade ‘3’ and were rated as rougher and breathier when compared to normal voice standards. TEP speech has been reported to have a “wet” quality in previous studies [5, 26, 38], that correlates with the “roughness” attribute in GRBAS. In our study, roughness was graded as medium in severity in the majority of our patients.

Arguably the most important outcome in speech restoration, intelligibility, was deemed poor overall compared with other studies that saw good outcomes in alaryngeal patients [8, 11], however, those evaluation methods were vaguely defined. Furthermore, our finding of poor intelligibility was at odds with patients all using the TEP as their preferred method of communication. Patients may have low expectations for the quality of their voice after reconstructive cancer surgery. A functional voice with the ability to communicate may be more important to these patients. Poor correlations between self-assessment and acoustic and perceptual dimensions in the assessment of highly irregular voices further supports the development of a standardized multidimensional approach to analysis [40].

There are several limitations to our study and the results should be interpreted in the context of its design. Firstly, this study was conducted at a single academic center with a relatively small sample of patients. Indeed, the number of patients might be low because we were limited by the number of surviving patients – most of who had hypopharyngeal cancers and/or recurrent cancers which carry inherently poor prognoses. Secondly, as has been recently editorialized, GPU is a complex procedure likely subject to a significant learning curve [41]. The patients treated in our cohort had GPU performed by a high-volume surgeon with considerable experience with the procedure, and this may limit the generalizability of the results presented. Thirdly, it is inherently difficult to analyze the sound of a substitute voice speaker. We tried to address this limitation by using several different outcome measures – objective acoustic analysis, subjective perceptual analysis, and a validated, self-reported measurement of voice - but recognize that even these measures may not be fully comprehensive. Fourthly, there was no comparison arm of normal patients or total laryngectomy patients with TEP. A sample size calculation revealed that the numbers needed to adequately power this statistical comparison would have been unfeasible. For a two-sided Students T Test at alpha = 0.05 and power = 0.8, with a minimal clinical difference in VHI-10 of 6 [42] as the primary outcome measurement, a sample size of 31 was needed in each group which could not feasibly be obtained for the GPU cohort. Fifthly, we were unable to perform any meaningful subgroup analysis of clinical factors predictive of voice outcomes following TEP in GPU because minimum event to variable thresholds could not be achieved using this small cohort of patients [43]. Still, this study represents the largest cohort of patients who underwent GPU and secondary TEP reported in the literature and demonstrates its safety and feasibility as well as the outcomes that can be achieved.

## Conclusion

Voice rehabilitation following laryngopharyngoesophagectomy and GPU reconstruction remains a significant challenge for patients. Secondary TEP is an option that is safe and feasible. Although patients demonstrated moderate subjective and objective impairment, TEP provided patients with a self-reported means of functional verbal communication and was their preferred method of communication.

## Data Availability

The datasets used and/or analysed during the current study are available from the corresponding author on reasonable request.

## Abbreviations

GPU: Gastric pull-up
TEP: Tracheoesophageal puncture
GRBAS: Grade roughness breathiness asthenia strain
VHI: Voice handicap index
IQR: Interquartile range
dB: Decibels
Hz: Hertz
TL: Total laryngectomy
f_0_: Fundamental frequency
XRT: Radiation therapy
HPx: Hypopharynx
Lx: Laryngeal
Tr: Trachea
E: Esophageal
Th: Thyroid
BOT: Base of tongue
VHI-10: Voice handicap index-10
